# Interferon signaling patterns in peripheral blood lymphocytes may predict clinical outcome after high-dose interferon therapy in melanoma patients

**DOI:** 10.1186/1479-5876-9-52

**Published:** 2011-05-05

**Authors:** Diana L Simons, Gerald Lee, John M Kirkwood, Peter P Lee

**Affiliations:** 1 Dept. of Medicine, Stanford University, Stanford, CA. USA; 2 Dept. of Medicine, University of Pittsburgh, Pittsburgh, PA. USA

**Keywords:** Melanoma, High-Dose Interferon, Lymphocyte Signaling, STAT1

## Abstract

**Background:**

High-dose Interferon (HDI) therapy produces a clinical response and achieves relapse-free survival in 20-33% of patients with operable high risk or metastatic melanoma. However, patients may develop significant side effects frequently necessitating dose reduction or discontinuation of therapy. We recently showed that peripheral blood lymphocytes (PBL) from some melanoma patients have impaired interferon (IFN) signaling which could be restored with high concentrations of IFN. This exploratory study evaluated IFN signaling in PBL of melanoma patients to assess whether the restoration of PBL IFN signaling may predict a beneficial effect for HDI in melanoma patients.

**Methods:**

PBL from 14 melanoma patients harvested on Day 0 and Day 29 of neoadjuvant HDI induction therapy were analyzed using phosflow to assess their interferon signaling patterns through IFN-α induced phosphorylation of STAT1-Y701.

**Results:**

Patients who had a clinical response to HDI showed a lower PBL interferon signaling capacity than non-responders at baseline (Day 0). Additionally, clinical responders and patients with good long-term outcome showed a significant increase in their PBL interferon signaling from Day 0 to Day 29 compared to clinical non-responders and patients that developed metastatic disease. The differences in STAT1 activation from pre- to post- HDI treatment could distinguish between patients who were inclined to have a favorable or unfavorable outcome.

**Conclusion:**

While the sample size is small, these results suggest that interferon signaling patterns in PBL correlate with clinical responses and may predict clinical outcome after HDI in patients with melanoma. A larger confirmatory study is warranted, which may yield a novel approach to select patients for HDI therapy.

## Background

High-dose Interferon (HDI) therapy produces a clinical response and achieves relapse-free survival in 20-33% of patients with operable high risk or metastatic melanoma [1-9]. However, patients may develop significant side effects frequently necessitating dose reduction or discontinuation of therapy. Therefore, approaches to select patients for initiation and/or maintenance on HDI therapy would be very useful.

While interferon has been shown to induce anti-tumor effects such as anti-proliferative, anti-vascular [10] and pro-apoptotic effects [11], it has also been suggested that HDI therapy mediates its effects through modulating the immune response [12]. Indeed development of autoimmunity [13] and a certain serum cytokine profile [14] have been shown to correlate with clinical responses in HDI adjuvant treated melanoma patients. Nonetheless, the mechanism of HDI's immunomodulatory roles is unclear and it is uncertain how these correspond with the autoimmune effects induced by IFN therapy.

We recently showed that peripheral blood lymphocytes (PBL) from patients with melanoma and other cancers have reduced phosphorylation of signal transducer and activators of transcription 1 (pSTAT1) upon Interferon-α (IFN-α) stimulation, demonstrating a defect in Type I IFN signaling [15,16]. Moreover, such defects could be partially restored by prolonged stimulation with IFN [15]. This offered a possible mechanism for the beneficial effect of HDI therapy in melanoma patients, and also suggested a way to select patients for therapy based on their PBL IFN signaling patterns.

Type I IFNs (α/β) have been recognized to have important and diverse immunoregulatory functions. These include promoting proliferation and clonal expansion of CD4 and CD8 T cells [17-20], enhancing antibody production of B cells [21,22], and increasing cytotoxic activity of natural killer cells (NK) and CD8 T cells [23,24]. IFN also has negative effects on the activation and proliferation of T regulatory cells (Tregs) [25], which are known for their immunosuppressive roles in cancer. With the advancement of flow cytometry-based assays, signaling profiles of immune cells can be measured with increased sensitivity through phospho-flow (phosflow) analysis, which provides the ability to concurrently measure signaling activities within multiple cell types.

In the present study, we measured IFN signaling responses in peripheral blood lymphocytes from stage IIIB-C melanoma patients taken before treatment and at day 29 of neo-adjuvant HDI therapy. In addition, all of these patients continued on a maintenance regimen of HDI post surgical resection. Archived peripheral blood mononuclear cells (PBMCs) were assessed using phosflow to measure Type I IFN signaling responses through IFN-α induced phosphorylation of STAT1-Y701 in patients undergoing HDI therapy with known short-term clinical responses and long-term clinical outcome. This exploratory study found that there was a correlation in PBL T cells between response to IFN-α induced STAT1 activation and clinical responses during the induction phase of HDI. Moreover, we were able to correlate STAT1 activation in T cells from HDI treated melanoma patients over the 4-week induction phase to clinical outcome, demonstrating that measuring the IFN signaling patterns in peripheral blood lymphocytes may be useful to select patients who are more likely to benefit from HDI maintenance therapy.

## Methods

### Patient Characteristics

Archived peripheral blood mononuclear cells (PBMCs) from 14 Stage IIIB-C melanoma patients (a total of 28 PBMC samples, 14 acquired pre- and 14 acquired post-HDI treatment) were analyzed for STAT1-Y701 phosphorylation (pSTAT1) levels by phosflow cytometry. Patient demographics and clinical details are shown in Table 1.

**Table 1:** Patient characteristics and clinical outcome of HDI treated melanoma patients

Patient ID	Age (y) *	Gender	Clinical Response	Status at Follow-up	HDI Completed	Duration of Disease Free (mo)	Current status
890	50	M	CR	MET	Y, DR	32	Deceased
901	62	F	NR	MET	Y	2	Deceased
903	45	M	PR	NED	Y	86	Alive
973	59	M	PR	NED	Y	54	Alive
974	70	F	NR	NED	Y, DRx2	67	Alive
978	75	M	PR	MET	Y	12	Deceased
980	56	F	NR	MET	Y, DRx2	6	Deceased
983	45	M	NR	MET	Y	6	Deceased
985	76	F	PR	NED	Y, DR	65	Alive
1006	78	M	PR	MET	Y	6	Deceased
1008	49	M	PR	NED	Y, DR	61	Alive
1015	57	F	PR	MET	Y	4	Deceased
1018	44	M	PR	NED	Y	60	Alive
1052	54	M	NR	MET	Y	19	Alive

NOTE: Adapted from ref.[12]

*Age at time of last contact.

HDI: High Dose IFN-α2b; CR: Complete Response; PR: Partial Response; NR: No Response; MET: Metastasis; NED: No Evidence of Disease; DR: Dose Reduction; DRx2: Two Dose Reductions

These patients participated in a clinical trial for neoadjuvant therapy with HDI and their treatment has been thoroughly described [12]. Briefly, the HDI induction phase consisted of IFN-α2b 20 million units (MU)/m^2 ^per day intravenously, 5 days per week (Monday to Friday) for 4 weeks. Following surgical resection, patients were placed on a HDI maintenance regimen consisting of IFN-α2b 10 MU/m^2 ^per day subcutaneously, three times per week (Monday, Wednesday, Friday) for 48 weeks. For each patient, blood samples were taken before (Day 0-pre) and after the 4-week induction phase of HDI (Day 29-post). Due to adverse events, 5 patients underwent 1/3 dose reductions and two of these patients required dose reductions twice (Table 1). Phosphorylated STAT1 levels were measured in lymphocytes, T cell subsets (both CD4 and CD8) and B cells with or without stimulation of IFN-α for each of these two time points. All patients signed informed consent and the study was approved by the University of Pittsburgh (Pittsburgh, PA) Institutional Review Board.

Interim responses were determined using WHO criteria [26] and patients were classified as clinical responders or non-responders based on a measure of tumor reduction both clinically and histologically over the 4-week induction phase of HDI therapy[12]. Among these patients, 9 showed a complete response (n = 1) or partial response (n = 8) and both were grouped into a single responder group (R). Five patients did not exhibit a clinical response and were grouped as non-responders (NR).

For long-term clinical outcome, patients were further classified as exhibiting no evidence of disease (NED) or metastatic disease (MET) based on their status at the time of follow-up (range 9-86 months) after completing a maintenance regimen of 48 weeks. Six patients were classified as NED and exhibited no evidence of metastases at a minimum follow-up of 4.5 years. In contrast, all of the 8 MET patients developed metastatic disease within 3 years and three of these were disease free for up to 1 year (Table 1).

### IFN-α Stimulation and Detection of pSTAT1-Y701 in HDI-treated Melanoma PBMCs

IFN-α stimulation and detection of pSTAT1-Y701 in cancer patients have been previously described [16] with modifications. Briefly, cryopreserved PBMCs were thawed and rested overnight in IMDM 10% FBS at 37°C 7% CO_2_. Cells were ficolled, resuspended to 2 × 10^6 ^cells per 50 μl in IMDM 5% human AB serum (HS) and stained with mouse anti-human CD3 FITC, CD8 PE-Cy7, CD4 PE-AF700 and CD19 PE-TR (Caltag-Invitrogen) for 30 minutes. IMDM 5%HS was added to each tube and 1 × 10^6 ^cells were aliquoted per test. PBMCs remained unstimulated or were stimulated with IFN-α (NIAID Reference Reagent Repository) to a final concentration of 1000 IU/ml and incubated at 37°C 7%CO_2 _for 15 minutes. Cells were fixed by formalin and incubated at 37°C 7% CO_2 _for 10 minutes. PBMCs were washed twice with 1× PBS, resuspended in 1 ml of 1× Custom Perm Buffer (#643435, BD Biosciences), and incubated for 30 minutes at room temperature. Cells were washed in wash buffer (1× PBS, 2% FBS, 0.09% sodium azide), resuspended to exactly 50 μl and incubated with mouse anti-human STAT1-pY701 Alexa Fluor^® ^647(BD Biosciences) for 1 hour at room temperature. Cells were washed in wash buffer and put on ice until analyzed by flow cytometry on a LSRII flow cytometer (BD Biosciences). Paired pre-and corresponding post- PBMC samples were assayed on the same day.

### Data and Statistical Analysis

Flow cytometry FCS files were analyzed using FlowJo 8.5.3 (Treestar, http://www.treestar.com). Gating strategy for the selection of lymphocytes, T cells and B cells are shown in Additional File 1 Figure S1. The mean fluorescent intensity (MFI) of STAT1-pY701 Alexa Fluor^® ^647 was calculated for all stimulated and unstimulated samples and fold changes were determined by dividing the MFI of stimulated samples by the MFI of the corresponding unstimulated samples. Basal levels of STAT1-Y701 were determined by the MFI in unstimulated cells. Kaplan-Meier survival curves were generated and the correlation of IFN-α induced pSTAT1 with disease-free and overall survival was estimated using the log-rank test. For the purpose of these comparisons, a ratio for each patients' lymphocytes were determined by dividing the fold change in pSTAT1 post-treatment by the fold change in pSTAT1 pre-treatment. A median of these ratios was generated using all patients in the study (n = 14) and patients were segregated according to whether they fell within a range of ± 0.1 around the median. Data was analyzed using Graphpad Prism 5.00 and the R statistical package 2.7.1 http://www.r-project.org. P-values, estimated differences and 95% confidence intervals were calculated with R software from the Comprehensive R Archive Network using nonparametric unpaired or paired two-sided Wilcoxon-Mann-Whitney T-tests. The False Discovery Rate (FDR) was calculated in R and used to adjust for multiple comparisons testing [27]. Adjusted P-values < 0.05 were considered significant. Coefficient of variations (CV) was calculated by dividing the standard deviation with the mean of the fold changes multiplied by 100.

## Results

### Differences in IFN responses between clinical responders and non-responders

**Figure 1:**
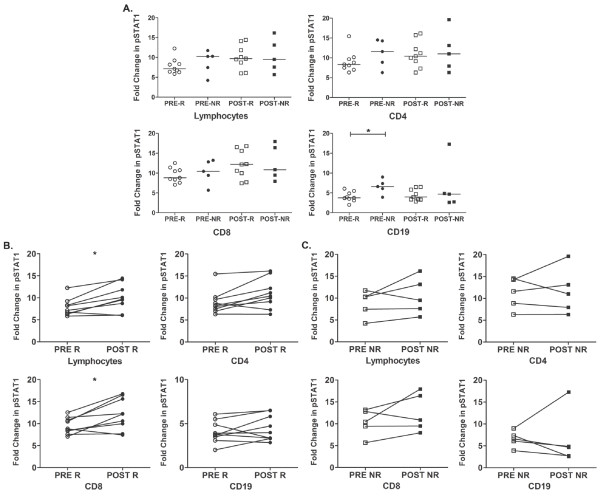
**IFN-α induced fold change of pSTAT1-Y701 in PBMCs from responding and non-responding patients**. PBMCs were stimulated with 1000 IU/ml of IFN-α or remained unstimulated and pSTAT1 was assessed by phosflow. The IFN-α induced fold change in pSTAT1 was measured in Lymphocytes, CD4 T cells, CD8 T cells and CD19 B cells. **A) **Unpaired analysis of PBMCs acquired before and after the 4 week induction phase with HDI were compared between responders (R: pre- open circle, post- open square) and HDI non-responders (NR: pre- closed circle, post- closed square). Two-sided Wilcoxon-Mann-Whitney unpaired analysis was used to compare between responding and non-responding lymphocytes and lymphocyte subsets. (* CD19: p = 0.028, 95% CI: 0.40 to 4.59, CV: pre-R 28.2%, pre-NR 30.9%). **B) **and **C) **Paired analysis of HDI responders (R) and HDI non-responders (NR) lymphocytes were assessed for their response to IFN-α through STAT1 activation before (pre-) and after (post-) the 4 week induction phase of HDI therapy. Two-sided Wilcoxon-Mann-Whitney paired analysis was used to compare response levels of pSTAT1 in responding and non-responding melanoma patients. The fold change was calculated by dividing the mean fluorescent intensity (MFI) of stimulated cells by the MFI of unstimulated cells. The median is indicated by the bar in each data set. Adjusted P-values < 0.05 were considered significant. CVs were calculated by dividing the standard deviation with the mean of the fold changes multiplied by 100. (* Lymphocytes: p = 0.039, 95% CI:-3.52 to -0.57, CV: pre-R 25.6%, post-R 30.1%; * CD8: p = 0.039, 95% CI: -5.0 to -0.43, CV: pre-R 19.6%, post-R 29.5%).

Initially, we compared STAT1-Y701 (pSTAT1) activation from patients who underwent IFN dose reductions to patients who did not undergo dose reductions (Table 1 data not shown) before and after the HDI induction phase. Both unpaired and paired analyses showed no significant changes in STAT1 activation between the patients who had dose reductions and patients who did not undergo dose reductions, and from day 0 to day 29 with HDI therapy. Subsequently, we addressed whether Type I IFN signaling differed between HDI clinical responders and clinical non-responders at day 0 or day 29 after HDI therapy. The induction of pSTAT1 from IFN-α stimulation was assessed by phosflow in PBMCs by examining the overall median fold change. The median fold change of IFN-α induced pSTAT1 in PBL from responders was lower than non-responders on day 0 (Figure 1A), which was observed in both CD4 and CD8 T cells, but these differences were not statistically significant. In CD19 B cells, a statistically significant difference was observed in the median fold change of pSTAT1 induced by IFN-α in responders and non-responders on day 0 (Figure 1A). The median fold change of pSTAT1 induced by IFN-α in lymphocytes and lymphocyte subsets showed little or no difference between responders and non-responders on day 29 (Figure 1A).

### Changes in Type I IFN responses from day 0 to day 29 in clinical responders and non-responders

**Figure 2:**
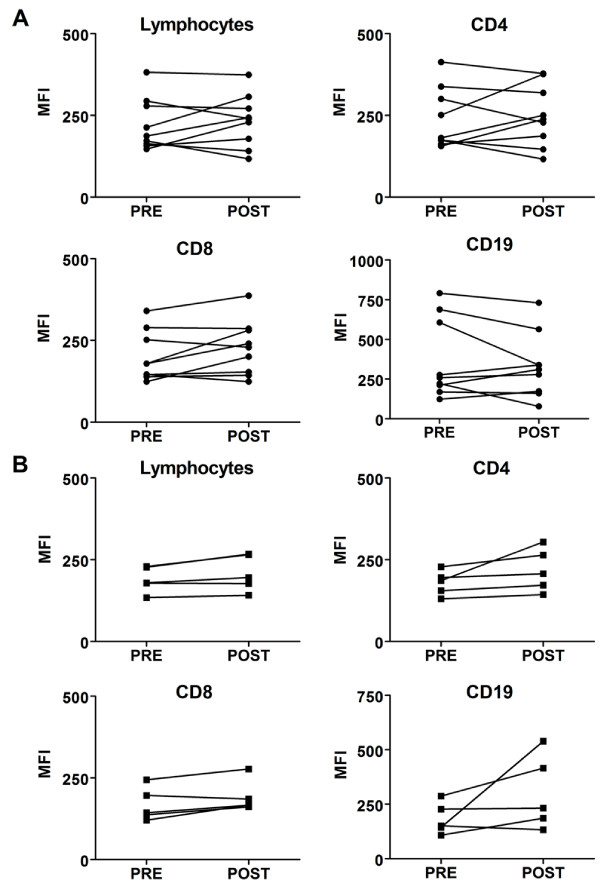
**Basal levels of pSTAT1-Y701 in PBMC subsets from HDI treated responders and non-responders**. Changes in basal response levels of pSTAT1 before (pre-) and after (post-) the 4 week induction phase with HDI were compared in Lymphocytes, CD4 and CD8 T cells, and CD19 B cells in HDI treated **A) **responding and **B) **non-responding melanoma patients. Two-sided paired Wilcoxon-Mann-Whitney tests were performed on melanoma patient's pre- and corresponding post- PBMCs. Adjusted P-values < 0.05 were considered significant.

We further investigated within the HDI-responding and non-responding patients on day 0 and day 29 and examined the effect of HDI therapy on Type I IFN signaling. Paired samples from pre-treated HDI PBMCs were compared to lymphocytes from their corresponding post-HDI treated PBMCs in both melanoma responding and non-responding patients. Two-sided Wilcoxon-Mann-Whitney paired analysis demonstrated that there was a statistically significant increase in STAT1 activation after HDI treatment in the responding group, and the response was equally observed in lymphocytes overall and in CD8 T cells (adjusted p-values 0.039, respectively). CD4 T cells were on the cusp of significance (adjusted p-value, 0.052) and B cells showed no significant differences in STAT1 activation (adjusted p-value, 0.30) (Figure 1B). In contrast, there were no significant differences in the response levels within any lymphocyte subset of non-responders from pre- to post-HDI treatment (Figure 1C). To determine whether the overall response was due to the basal levels of pSTAT1, we analyzed changes in pSTAT1 in PBLs in unstimulated cells from before to after HDI treatment. We found no significant differences in the basal expression of pSTAT1 from pre to post HDI treatment in both the responding and non-responding patients (Figure 2A-B).

### IFN signaling patterns and clinical outcome

**Figure 3:**
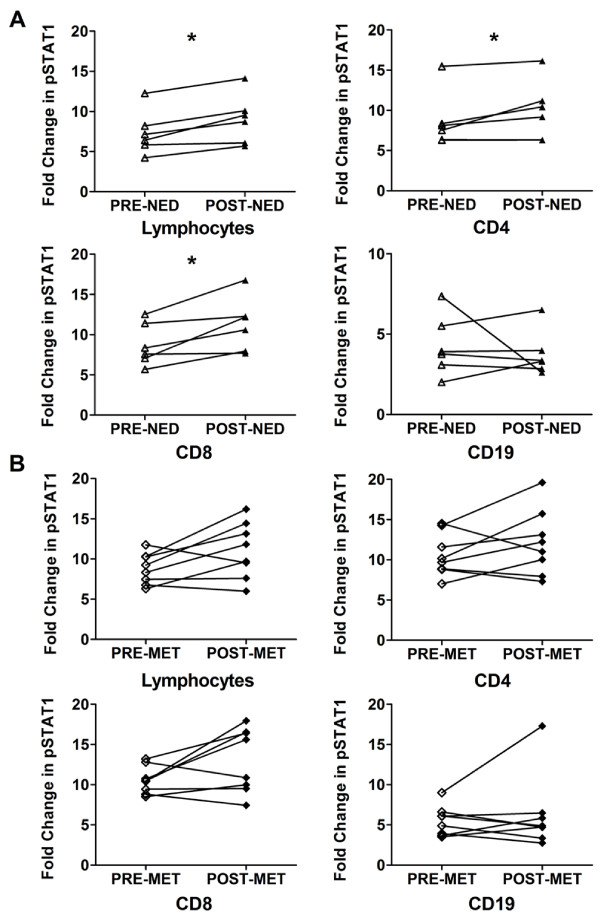
**Correlation of IFN-α induced pSTAT1 and long-term clinical outcome in HDI patients**. At the time of clinical follow-up, patients were classified as exhibiting no evidence of disease (NED) or metastases (MET). The IFN-α induced fold change of pSTAT1 in melanoma patients' lymphocytes and lymphocyte subsets were assessed in patients exhibiting **A) **NED and, **B) **MET before (pre-) and after (post-) the HDI induction phase. Two-sided paired Wilcoxon-Mann-Whitney tests were performed on melanoma patients pre- and corresponding post- PBMCs and adjusted P-values < 0.05 were considered significant. CVs were calculated by dividing the standard deviation with the mean of the fold changes multiplied by 100. (*Lymphocytes: p = 0.042, 95% CI: -3.12 to -0.25, CV: pre-NED 37.4%, post-NED 34%; *CD4: p = 0.042, 95% CI: -4.77 to -0.48, CV: pre-NED 39.6%, post-NED 36.9%; *CD8: p = 0.042, 95% CI: -5.12 to -0.13, CV: pre-NED 30.2%, post-NED 29.9%).

It was observed that disease-free and overall survival was longer amongst patients with a clinical response at day 29 compared with non-responders, although the results did not reach statistical significance [12]. We investigated the association of long-term clinical outcome with IFN-α induced pSTAT1 levels in HDI treated melanoma patient lymphocytes from Day 0 to Day 29. Paired-analysis was used to compare patients who showed no evidence of disease (NED) or who developed subsequent metastatic disease (MET) at the time of clinical follow-up. NED patients demonstrated a significant increase in the activation of STAT1 from Day 0 to Day 29 in lymphocytes and both CD4 and CD8 T cells (adjusted p-values, 0.04 [Figure 3A, Additional File 2 Figure S2]). In contrast, lymphocytes from MET patients did not show a consistent increase in the induction of pSTAT1 from IFN-α stimulation from pre- to post-HDI therapy (Figure 3B).

**Figure 4:**
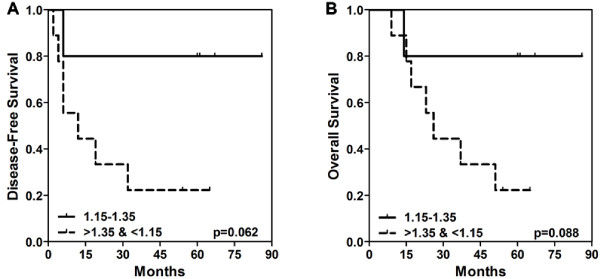
**Disease-free and overall survival analysis in melanoma patient lymphocytes**. Kaplan-Meier survival curves were generated to assess the correlation of STAT1 activation with **A) **disease-free survival (p = 0.062) and, **B) **overall survival (p = 0.088). A ratio for each patient was calculated by dividing the fold induction of pSTAT1 in post-treated lymphocytes by the fold induction of pSTAT1 in pre-treated lymphocytes (post/pre). A median of these ratios was generated (1.25) using all patients in the study (n = 14) and patients were segregated according to whether they fell within a range of ± 0.1 around the median. P-values < 0.05 were considered significant.

Since STAT1 activation correlated with long-term clinical outcome, we further examined pSTAT1 responses in lymphocytes with disease-free and overall survival. A ratio for each patient's lymphocytes was determined by dividing the fold change in pSTAT1 post-treatment by the fold change in pSTAT1 pre-treatment. A median of these ratios (1.25) was generated using all patients in the study (n = 14) and patients were segregated according to whether they fell within a range of ± 0.1 around the median. Patients whose pSTAT1 ratios fell within this range had better disease-free and overall survival (Figure 4A-B, respectively) as compared to patients who had minimal or negative signaling changes (median <1.15), and interestingly, also patients who had larger increases from pre- to post- HDI treatment (median >1.35).

## Discussion

Previously, we have demonstrated in two independent cohorts impaired IFN signaling and downstream functional consequences in PBLs from patients with minimally metastatic stage III and widely metastatic stage IV melanoma as compared to healthy controls [15,16]. In the current study, we analyzed serial PBMC samples obtained from patients before and after the induction phase of HDI in a new independent cohort of minimally metastatic stage IIIb and IIIc melanoma patients to advance our current understanding of immune dysfunction in cancer. This exploratory study demonstrated that patients with high-risk operable nodal involvement with melanoma who had a clinical response to high dose IFN-α2b therapy over the 4-week induction phase of neoadjuvant therapy had a significant increase in STAT1 activation in peripheral blood T cells, but not B cells, upon IFN-α stimulation from Day 0 to Day 29. Moreover, this increase in pSTAT1 in peripheral blood T cells also correlated with good clinical outcome suggesting the efficacy of HDI in the clinical responders may be due, as least in part, to augmentation of their pSTAT1 responsiveness.

Moreover, the differences in STAT1 activation from pre- to post- HDI treatment could distinguish patients who were inclined to have a favorable or unfavorable outcome. As expected, patients who had minimal or negative changes in pSTAT1 (median ratio < 1.15) had poor outcome. Of patients who showed increased pSTAT1 signaling after HDI therapy, only patients who displayed modest augmentation (median ratio 1.15 - 1.35) had good outcome. Interestingly, patients who had 'hyper' IFN signaling responses (median ratio >1.35) of pSTAT1 pre- to post- HDI therapy had poor outcome, similar to those who has minimal or negative changes. These results warrant further confirmation in a larger patient cohort to investigate the underlying mechanisms by which HDI alters IFN signaling patterns in patients with different clinical outcomes.

Assessing the IFN signaling patterns in peripheral blood T cells from melanoma patients from Day 0 to Day 29 HDI therapy may be a clinically useful approach to select patients who would be more inclined to benefit from further treatment, and hence should be maintained on HDI. A trend was observed which showed that responding patients, prior to HDI therapy, have a lower response to IFN-α induced pSTAT1 compared to those of the non-responding patients. We have previously found two subsets of IFN responses in melanoma patients, IFN low-responders and IFN high-responders [15]. We showed that in IFN low-responders, prolonged in vitro stimulation with high doses of IFN partially restored IFN signaling, suggesting a possible mechanism for the beneficial effect of HDI therapy in these melanoma patients. Prior to initiation with HDI (pre), reduced activation of STAT1 in the responding patients compared to the non-responding patients may be explained in that this patient subset exhibited a severe impairment in IFN signaling which was restored during the initiation phase of HDI therapy. In contrast, patients who had higher levels of STAT1 activation prior to the HDI initiation phase may not have had an IFN signaling defect and therefore, would not have benefited from HDI therapy. In our previous study [16], the differences in the median activation of pSTAT1 between melanoma patients and healthy controls were 1.6 fold were as, in the current study, the median differences in STAT1 activation between the responders and non-responders were 1.4 fold.

Though B cells showed a significantly lower trend in overall STAT1 activation in responders compared to non-responders before HDI, there was no significant increase in STAT1 activation during the induction phase of HDI in the responding group, and interestingly, non-responders exhibited decreased overall STAT1 activation. It has been reported that subsets of immune cells respond differently to IFNs [28,29] and that in the presence of high amounts of exogenous IFNs, downregulation of the receptors may occur as a negative feedback mechanism [30], thereby reducing responsiveness to IFNs. Additionally, reduced responsiveness in leukocytes may reflect the effects of a high tumor burden whereby tumor cells secreting immunosuppressive cytokines, such as IL-10 and TGF-β, have been shown to induce expression of STAT1 negative regulators such as the suppressors of cytokine signaling (SOCS) proteins and Src homology 2 (SH2)-containing phosphatase-1 and -2 and CD45 [31-33], thereby inhibiting the anti-tumor activity of immune effector cells [34]. Tregs and myeloid-derived suppressor cells, known for their suppressive roles on immune cells [35-37] in cancer, may also contribute to reduced responsiveness to HDI. Altered plasma or serum cytokine profiles in cancer patients [38,39] may predispose peripheral blood leukocytes to impaired IFN signaling.

There was variation and overlap between the responder and non-responder groups. We and others have previously described variation of signaling responses in melanoma patients' PBMCs using phosflow [15,40,41], suggesting that signaling abnormalities may not arise in all patients, but rather in a subset of patients. Variable responsiveness may explain the small differences observed between the clinical responders and non-responders. The sample size of this study was too small (14 patients) to be conclusive, but these results warrant a larger confirmatory study.

The importance of STAT1 in IFN signaling has been demonstrated in STAT1 knockout mice where STAT1 deficient mice were more likely to develop spontaneous tumors than wild type mice [42], and were more susceptible to viruses and pathogens showing an IFN-dependent link to STAT1 [43]. Previous studies have attempted to link pSTAT1 (and pSTAT3) levels in tumor cells and lymphocytes to clinical outcome of melanoma patients receiving interferon treatment [44,45]. Patients with higher pSTAT1/pSTAT3 ratios in pretreated lymph node biopsy tissues had better clinical outcome [44]; however, these studies did not find a correlation between pSTAT1/pSTAT3 ratios among lymphocytes of regional lymph nodes and survival. One novelty in the present study is to consider pSTAT1 levels in different subsets of peripheral blood lymphocytes.

The use of immune profiles as a prognostic tool to determine melanoma patient survival has been studied, such as using quantification of tumor infiltrating lymphocytes (TILs) in metastatic lesions [46], as well as gene expression profiling of TILs and CD3 T cells from primary cutaneous melanoma where the genes that were positively associated with survival were mainly related to the immune response [47]. Beyond these prognostic implications, assessing STAT1 activation in PBL of melanoma patients may provide an additional predictive tool to guide the application of HDI therapy.

## Conclusion

While the sample size was size, we have found encouraging results which point to measuring STAT1 activation in PBL T cells from Stage IIIB-C melanoma patients to stratify patients according to their potential to benefit from HDI. These results build upon prior studies of patients with advanced melanoma, and warrant confirmatory studies with a larger cohort of melanoma patients who are to receive HDI therapy. This is currently planned in the context of new intergroup studies of HDI. Other agents that enhance IFN signaling in T cells may be developed as novel therapy for melanoma, especially those that do not have the side effects of HDI.

## Competing interests

The authors declare that they have no competing interests.

## Authors' contributions

PL and JK designed the study. JK provided the clinical samples. DS and GL carried out the experiments. DS, GL, JK, and PL analyzed the results, and wrote the manuscript. All authors read and approved the final manuscript.

## Supplementary Material

Additional file 1 **Figure S1. Gating of lymphocytes, T cells and B cells for phosflow analysis**. **A) **Lymphocytes were gated based on their FSC and SSC properties. **B) **Within the lymphocyte gate, B cells were selected by gating on CD19+CD3- events and T cells were selected by gating on CD3+CD19- events. **C) **T cells were further divided into CD4+CD8- T helper cells and CD4-CD8+ cytotoxic T cells. **D) **Phosphorylation of STAT1-Y701 is demonstrated in stimulated cells (blue line) versus unstimulated cells (red line). Click here for file

Additional file 2 **Figure S2. IFN-induced pSTAT1 expression in lymphocytes**. Histogram overlays were generated for unstimulated (thin black line) and IFN-α stimulated (bold black line) lymphocytes for A) NED and B) MET patients before HDI therapy (open histograms) and after (shaded histograms) 29 days with HDI therapy. * Indicates melanoma patients that were clinical non-responders. CVs were calculated by dividing the standard deviation with the mean of the fold changes multiplied by 100. CV: pre-NED 37.4%, post-NED 34%, pre-MET 21.8%, post-MET 31.5%. Click here for file
