# Supplementary figures and images for: Interferon signaling patterns in peripheral blood lymphocytes may predict clinical outcome after high-dose interferon therapy in melanoma patients
Source: J Transl Med. 2011 May 5;9:52. doi: 10.1186/1479-5876-9-52 (PMC3114759; doi:10.1186/1479-5876-9-52)

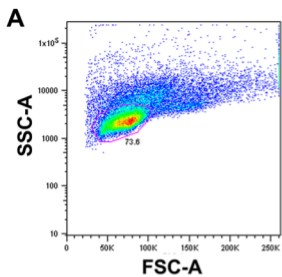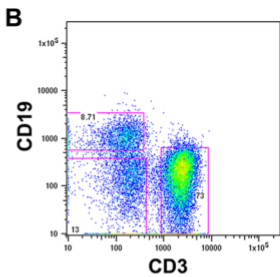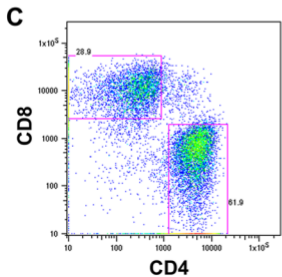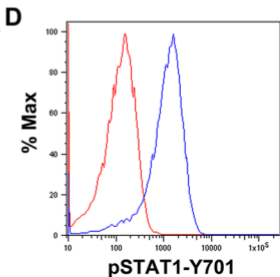

Supplement: Additional file 1 — Figure S1. Gating of lymphocytes, T cells and B cells for phosflow analysis. A) Lymphocytes were gated based on their FSC and SSC properties. B) Within the lymphocyte gate, B cells were selected by gating on CD19+CD3- events and T cells were selected by gating on CD3+CD19- events. C) T cells were further divided into CD4+CD8- T helper cells and CD4-CD8+ cytotoxic T cells. D) Phosphorylation of STAT1-Y701 is demonstrated in stimulated cells (blue line) versus unstimulated cells (red line). [file 1479-5876-9-52-S1.PDF]

**A.**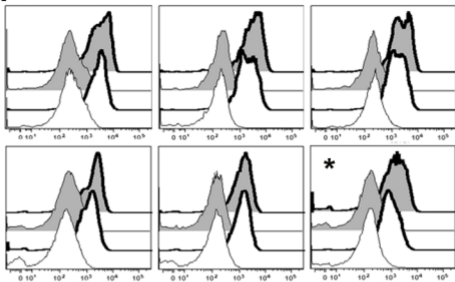**B.**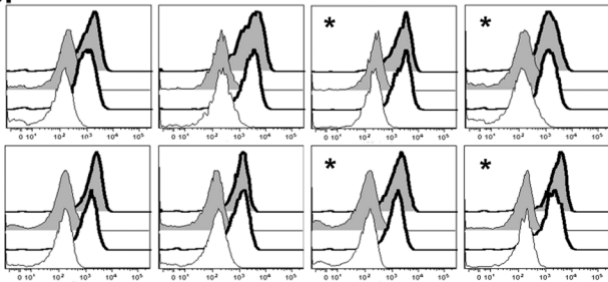**pSTAT1-Y701**

Supplement: Additional file 2 — Figure S2. IFN-induced pSTAT1 expression in lymphocytes. Histogram overlays were generated for unstimulated (thin black line) and IFN-α stimulated (bold black line) lymphocytes for A) NED and B) MET patients before HDI therapy (open histograms) and after (shaded histograms) 29 days with HDI therapy. * Indicates melanoma patients that were clinical non-responders. CVs were calculated by dividing the standard deviation with the mean of the fold changes multiplied by 100. CV: pre-NED 37.4%, post-NED 34%, pre-MET 21.8%, post-MET 31.5%. [file 1479-5876-9-52-S2.PDF]
